# Prevalence of dental caries in the permanent dentition amongst 12-year-olds in Africa: a systematic review and meta-analysis

**DOI:** 10.1186/s12903-022-02489-4

**Published:** 2022-10-24

**Authors:** F. Kimmie-Dhansay, A. Bhayat

**Affiliations:** 1 Department of Community Oral Health, University of the Western Cape, Cape Town, South Africa; 2 Department of Community Dentistry, University of Pretoria, Pretoria, South Africa

**Keywords:** DMFT, 12-year-old, Caries prevalence, Africa, Urban, Rural

## Abstract

**Background:**

Dental caries (DC) is highly prevalent condition affecting mostly young children. There has been no systematic review done on the prevalence of DC amongst 12-year -olds in Africa. Although some African countries have reported a decrease in DC prevalence, others have shown an increase and it is essential to measure current trends in order to identify strategies and programmes that could assist in reducing DC in Africa. The aim of this systematic review was to determine the prevalence of DC (condition) amongst the permanent dentition of 12-year-old children (population) in Africa (context).

**Methods:**

A systematic review and meta-analysis was performed. Peer reviewed cross-sectional articles from January 2000 until December 2021 was searched and this included the following databases: Pubmed (Medline); SCOPUS; CINAHL (via EBSCOhost); Academic Search Complete (via EBSCOhost); Dentistry and Oral Sciences Sources (via EBSCOhost); and Science Direct. The search was last updated on the 10th January 2022. Joanna Briggs Institute critical appraisal tools were used to assess risk of bias. Prevalence figures were stratified by Urban/Rural status, country and time using a random-effects model. All studies performed on children 12-year-olds on the African continent were included. The prevalence of DC and the DMFT scores were the primary and secondary outcome measures, respectively. Only articles consisting of 12-year-old children who reside in Africa were included in this study. The systematic review was registered with Prospero CRD42021293666.

**Results:**

18,080 participants were included in this review. A total of thirty studies were included in the review. The pooled effect size of dental caries severity was 1.09 (CI 0.91–1.27) and the overall prevalence was 36% (CI 29.4–41.7%). Eritrea (78%) had the highest prevalence of DC while Zambia had the lowest (11%); Eritrea also had the highest DMFT score (2.5) with Sudan having the lowest score (0.49). Urban cities had the highest DMFT score (1.32, CI 0.97–1.68), compared to rural cities (1.13, CI 0.86–1.4) and there was an increasing trend in DC prevalence over time from 28% (CI 23–34%) in 2000 to 2005 to 57% (CI 43–72%) in studies conducted after 2015. The risk of bias was very low where majority of the studies scored more than 50% in the JBI critical appraisal tool.

**Conclusions:**

There was a wide discrepancy in the DC prevalence and scores across the different countries, settings (rural versus urban) and there was an increase in the prevalence over time. This review was self-funded.

## Introduction

Dental caries (DC) in children is a highly prevalent disease often resulting in pain and difficulty in mastication [1]. According to the Global Burden of Disease study, the prevalence of untreated DC in permanent teeth is 267 million [2].

The sequelae of untreated DC in children include poor school performance [3, 4]; high school absenteeism [3]; verbal bullying [5] and has shown to impact the Oral Health Related Quality of Life (OHRQoL) [6]. The prevalence of caries in 12-year-olds varies from country to country and even within continents. In Africa, the prevalence ranged from 42 to 78% [7–12].

DC share risk factors associated with obesity [14, 15]. Although aetiological factors have been described well in literature [16–18], there is still a very high prevalence rate across the globe. There are significant costs are associated with the management of DC and preventative measures, such as toothbrushing, are a much cheaper alternative [19]. However oral health literacy remains quite low [20].

Prevalence of DC in 12-year-olds have been reported in systematic review which highlighted that caries prevalence ranged between 41.9 and 69.4% in lower-middle and upper middle income countries [13]. A study conducted in Dominican Republic and Lithuania reported prevalence of 73% [21] and 85.5% [22]. However, an overall view of dental caries prevalence in 12-year-olds in Africa has not been conducted.

The aim of the systematic review was to determine the prevalence of dental caries (condition) in 12-year-olds (population) in Africa (context) using cross-sectional study designs. The results could assist in identifying public health programmes that can be implemented to manage and reduce the prevalence of DC.

## Methodology

The Meta-Analysis of Observational Studies in Epidemiology (MOOSE) guideline was used to conduct this study [23]. A comprehensive search strategy was developed by a community dentistry specialist, a biostatistician and a clinical epidemiologist with a very good background in search strategy. Studies only in English were included. The authors (FKD and AB) conducted a pilot search strategy together before finalizing the ultimate search strategy that was going to be utilized for this study. Searching peer-reviewed articles from January 2000 until December 2021 was conducted and this included the following databases: Pubmed (Medline); SCOPUS; CINAHL (via EBSCOhost); Academic Search Complete (via EBSCOhost); Dentistry and Oral Sciences Sources (via EBSCOhost); and Science Direct. The search strategy for the African studies was obtained from a previous publication [15] and the terms used are listed in Table 1. Contact with authors was documented in the design of this study, but not performed as it was not deemed necessary.

**Table 1:** Medical Subject Headings (MeSH) terms and Title/Abstract used 10th January 2022

Terms related to caries	Caries OR decay OR dmft OR dental OR oral OR ICDAS OR DMFT OR caries[Title/Abstract] OR decay[Title/Abstract] OR dmft[Title/Abstract] OR dental[Title/Abstract] OR oral[Title/Abstract] OR ICDAS[Title/Abstract] OR DMFT[Title/Abstract]))	1,685,869
Terms related to prevalence	Prevalence[Title/Abstract]) OR (prevalence)	837,230
Terms related to location of study	(“Africa”[MeSH] OR Africa*[tw] OR Algeria[tw] OR Angola[tw] OR Benin[tw] OR Botswana[tw] OR “Burkina Faso”[tw] OR Burundi[tw] OR Cameroon[tw] OR “Canary Islands”[tw] OR “Cape Verde”[tw] OR “Central African Republic”[tw] OR Chad[tw] OR Comoros[tw] OR Congo[tw] OR “Democratic Republic of Congo”[tw] OR Djibouti[tw] OR Egypt[tw] OR “Equatorial Guinea”[tw] OR Eritrea[tw] OR Ethiopia[tw] OR Gabon[tw] OR Gambia[tw] OR Ghana[tw] OR Guinea[tw] OR “Guinea Bissau”[tw] OR “Ivory Coast”[tw] OR “Cote d’Ivoire”[tw] OR Jamahiriya[tw] OR Jamahiryia[tw] OR Kenya[tw] OR Lesotho[tw] OR Liberia[tw] OR Libya[tw] OR Libia[tw] OR Madagascar[tw] OR Malawi[tw] OR Mali[tw] OR Mauritania[tw] OR Mauritius[tw] OR Mayote[tw] OR Morocco[tw] OR Mozambique[tw] OR Mocambique[tw] OR Namibia[tw] OR Niger[tw] OR Nigeria[tw] OR Principe[tw] OR Reunion[tw] OR Rwanda[tw] OR “Sao Tome”[tw] OR Senegal[tw] OR Seychelles[tw] OR “Sierra Leone”[tw] OR Somalia[tw] OR “South Africa”[tw] OR “St Helena”[tw] OR Sudan[tw] OR Swaziland[tw] OR Tanzania[tw] OR Togo[tw] OR Tunisia[tw] OR Uganda[tw] OR “Western Sahara”[tw] OR Zaire[tw] OR Zambia[tw] OR Zimbabwe[tw] OR “Central Africa”[tw] OR “Central African”[tw] OR “West Africa”[tw] OR “West African”[tw] OR “Western Africa”[tw] OR “Western African”[tw] OR “East Africa”[tw] OR “East African”[tw] OR “Eastern Africa”[tw] OR “Eastern African”[tw] OR “North Africa”[tw] OR “North African”[tw] OR “Northern Africa”[tw] OR “Northern African”[tw] OR “South African”[tw] OR “Southern Africa”[tw] OR “Southern African”[tw] OR “sub Saharan Africa”[tw] OR “sub Saharan African”[tw] OR “subSaharan Africa”[tw] OR “subSaharan African”[tw]) NOT (“guinea pig”[tw] OR “guinea pigs”[tw] OR “aspergillus niger”[tw])	584,549
Terms related to Children	((Child*[Title/Abstract]) OR ("Child*"))	2,728,820
Combination of terms		1539

We included children aged 12 years (plus or minus a year) [population], who had dental caries [condition] and who lived in Africa [context].

The search strategy for the African studies was obtained from a previous publication [24].

Hand-searching of eligible articles was also performed. All eligible articles were uploaded into Rayyan where all duplicate articles were removed [25].

### Screening and selection criteria

Two authors (FKD and AB) screened titles and abstracts, independently. Full text selection was performed independently. If any disagreements were found in abstract or full text selection, they were discussed until consensus was reached. Only studies conducted in Africa on children who were 12-year-olds (give or take a year) were included in this review. If either the number of children with caries, or the number of the complete sample was missing, then the articles were not included for the prevalence component of the study. If the number of the complete sample, the mean and standard deviation of the mean DMFT was not included, then the article was also not included in the DC severity component of the study. The following articles were excluded: articles without the full text, dissertations, articles not published in English, conference proceedings, letters to the editor, grey literature, and published protocols. Based on inclusion and exclusion criteria, articles were sorted in Rayyan [25] and any disagreements between the authors were clarified through discussion.

### Data extraction

Two authors (FKD and AB) extracted the data independently, if there were any disagreements, a consensus was reached through discussion. Although not necessary, the corresponding author could be contacted for any pertinent missing information from any included articles.

Author, year of publication, country of publication, study design, urban/rural status, diagnostic criteria, and the mean number of decayed, missing, and filled permanent teeth (DMFT) were recorded in Excel and uploaded into STATA for further examination.

### Critical appraisal

The Joanna Briggs Institute (JBI) critical appraisal checklist for studies reporting prevalence data was used to determine the quality of the included studies [26]. The critical appraisal was performed independently by the two authors and any disagreements were discussed until a consensus was reached. There are nine criteria in the JBI critical appraisal tool and a maximum score of nine indicated a lower risk of bias.

### Data synthesis

StataCorp. 2019. STATA Statistical Software: Release 17, College Station, TX: StataCorp LLC was used to conduct the meta-analyses. The I^2^ test and the Q test was used to determine statistical heterogeneity and subsequently random effects model was utilized due to the significant heterogeneity (I^2^ > 50%) results obtained. In addition the random /fixed effects model was chosen based on the Tufanaru article [27] which states that a minimum of 5 articles be used to run a random effects meta-analysis. Subgroup analysis was conducted for year of publication, urban/rural status, and country and the confidence intervals were set at 95%. A spatial representation of the distribution of pooled prevalence was carried out in QGIS software. Where possible subgroup analysis was performed per country, urban/rural status and year of publication. If the urban or rural status was unclear, a new category called, “urban/rural” was created.

## Results

### Search and selection

**Fig. 1:**
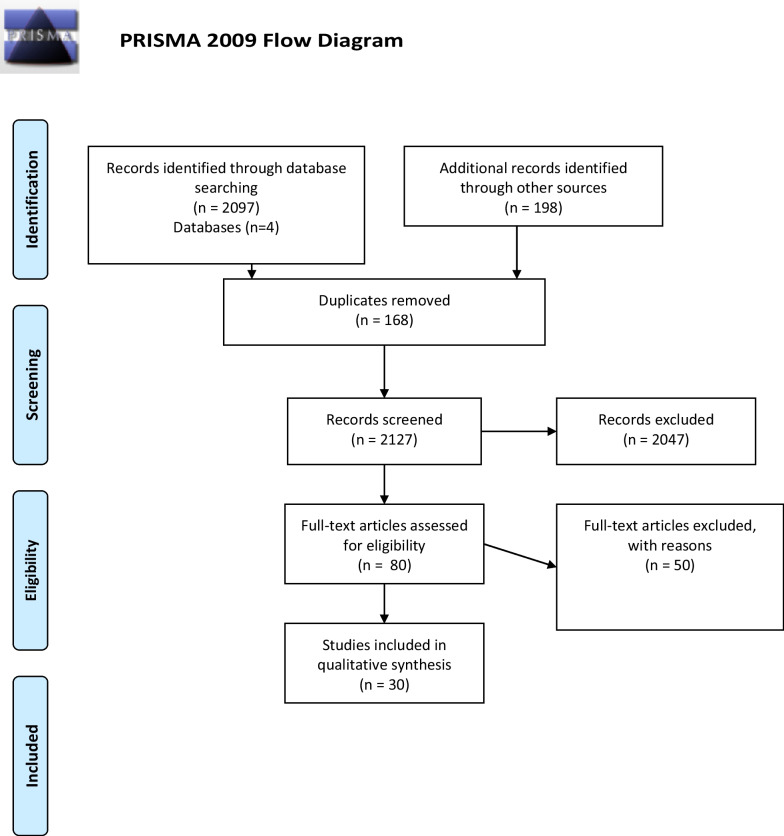
Flow diagram of article selection process

A total of 2097 articles, and a further 198 possible articles were identified before duplication was removed. After the 168 duplicate articles were removed, 2127 articles were screened, where a further 2047 articles were excluded by title and abstract. Thereafter, 80 articles were assessed by reading the full text for eligibility as described in the flow diagram (Fig. 1). Of these 80 articles, 30 articles met the criteria and were included. A total of 50 articles were excluded after reading the full texts and the reasons for their exclusion is shown in Table 2.

**Table 2:** List of articles excluded with reasons (n = 50)

Title	Author, year	Reasons for Exclusion
Dental Caries and Nutritional Status of School Children in Lagos, Nigeria-a Preliminary Survey Caries	Adeniyi [28]	Wrong age
The prevalence of dental caries among Egyptian children and adolescences and its association with age, socioeconomic status, dietary habits and other risk factors. A cross-sectional study	Abbass [29]	
Oral health in Tunisia	Abid [30]	Secondary data
Prevalence and pattern of dental caries among a sample of Nigerian public primary school children	Adeniyi [31]	Combined age groups
Prevalence of dental caries: national pilot study comparing the severity of decay (CAO) vs ICDAS index in Senegal	Aidara [32]	
Sociobehavioural risk factors of dental caries among selected adolescents in Ibadan, Nigeria	Ajayi [33]	
Assessment of dental caries status among school children aged 9–12 years of Sebha city-Libya by using of dft/ DMFT & SiC indices	Arrish [34]	
Caries experience and caries predictors–a study of Tanzanian children consuming drinking water with different fluoride concentrations	Awadia [35]	
Relating dental caries experience with body mass index among Nigerian primary school children: A cross-sectional survey	Azodo [36]	
Fluorosis, caries and oral hygiene in schoolchildren on the Ombili foundation in Namibia	Berndt [37]	
Severity of dental caries among 12-year-old Sudanese children with different fluoride exposure	Birkeland [38]	Wrong year of publication
Prevalence of oral conditions and associated factors among schoolchildren in Accra, Ghana: a cross-sectional study	Blankson [39]	Combined age groups
Dental caries, and supragingival plaque and calculus among students, Tanga, Tanzania	Carneiro [40]	
Caries and dental erosion: Are Soroti children and adolescents at risk from increased soft-drink availability in Uganda?	Cheng [41]	Wrong age
Dental caries and fluorosis among children in Lebanon	Doumit, 2018 [42]	Wrong population
Prevalence of dental caries and its impact on the academic performance of Sudanese basic school children, Al-Sahafa residential area (2013–2014)	El-Sayed, 2015[43]	Combined age groups
Relationship between oral health status of parents and that of their children	Faye [44]	Wrong language
Is chronotype profile a risk indicator for caries in children and adolescents in sub-urban Nigeria?	Folayan [45]	Secondary data
General anxiety, dental anxiety, digit sucking, caries and oral hygiene status of children resident in a semi-urban population in Nigeria	Folayan [46]	Combined age groups
Association between family structure and oral health of children with mixed dentition in suburban Nigeria	Folayan [47]	
Association between adverse childhood experiences, bullying, self-esteem, resilience, social support, caries and oral hygiene in children and adolescents in sub-urban Nigeria	Folayan [48]	
Dental Caries Experience among 6–14 years old Schoolchildren in Municipality of Tripoli Center, Libya	Gabroun [49]	
Dental fluorosis and caries experience in relation to three different drinking water fluoride levels in South Africa	Grobler [50]	
Dental caries, gingival health and malocclusion in 12-year-old urban Black schoolchildren from Soweto, Johannesburg	Hirschowitz [51]	Wrong year of publication
Using an oral health-related quality of life measure in three cultural settings	Hobdell [52]	Combined age groups
Self-reported dental pain and associated factors in Ugandan schoolchildren	Kiwanuka [53]	
Digit sucking habit and association with dental caries and oral hygiene status of children aged 6 months to 12 years resident in semi-urban Nigeria	Kolawole [54]	
Dental Caries Status among 6–14 Years Old School Going Children of Sebha city, Libya	Kumar [55]	
Impact of Untreated Dental Caries on Daily Performances of Children From Low Social Class in an Urban African Population: The Importance of Pain	Lawal [56]	Wrong population
Dental pain, oral impacts and perceived need for dental treatment in Tanzanian school students: A cross-sectional study	Mashoto [57]	Combined age groups
Discriminative ability of the generic and condition-specific Child-Oral Impacts on Daily Performances (Child-OIDP) by the Limpopo-Arusha School Health (LASH) project: a cross-sectional study	Mbawalla [58]	
Caries experience among school children in Enugu, Nigeria	Okoye [59]	
Variation in caries experience and sugar intake among secondary school students in urban and rural Uganda	Okullo [60]	
Disparities in caries experience and socio-behavioural risk indicators among private school children in Lagos, Nigeria	Olatosi [61]	
Dental caries experience and molar-incisor hypomineralisation in children: Pattern and severity	Oyedele [62]	
Impact of oral hygiene and socio-demographic factors on dental caries in a suburban population in Nigeria	Oyedele [63]	Secondary data
Assessment of dental caries and oral health challenges of school-age children in Rhino Camp Refugee Settlements in Arua, Uganda	Robert [64]	Combined age groups
Dental treatment needs among children and adolescents residing in an Ugandan Orphanage	Rubin [65]	
A longitudinal study of occlusal caries among schoolchildren in Dar es Salaam, Tanzania	Rugarabamu [66]	
Oral impacts on daily performances and its socio-demographic and clinical distribution: a cross-sectional study of adolescents living in Maasai population areas, Tanzania	Simangwa, 2020[67]	
Oral diseases and socio-demographic factors in adolescents living in Maasai population areas of Tanzania: a cross-sectional study	Simangwa [68]	
Dental caries on permanent dentition in primary school children	Simushi [69]	Not enough data
Changes in the prevalence of dental caries in primary school children in Lagos State, Nigeria	Sofola [70]	Combined age groups
Oral health status, knowledge of dental caries aetiology, and dental clinic attendance: A comparison of secondary school students in the rural and urban areas of Lagos	Soroye [71]	
Oral hygiene practices and caries prevalence among 9–15 years old Ghanaian School children	Ndanu [72]	
Prevalence of dental caries and associated factors among Finote Selam Primary School students aged 12–20 years, Finote Selam Town, Ethiopia	Teshome [73]	
Epidemiological profile of patients utilising public oral health services in Limpopo province, South Africa	Thema [74]	
Trends in dental caries prevalence, severity and unmet treatment need levels in South Africa between 1983 and 2002	van Wyk [75]	Secondary data
Baseline survey of oral health of primary and secondary school pupils in Uganda	Wandera [76]	Combined age groups

**Fig. 2:**
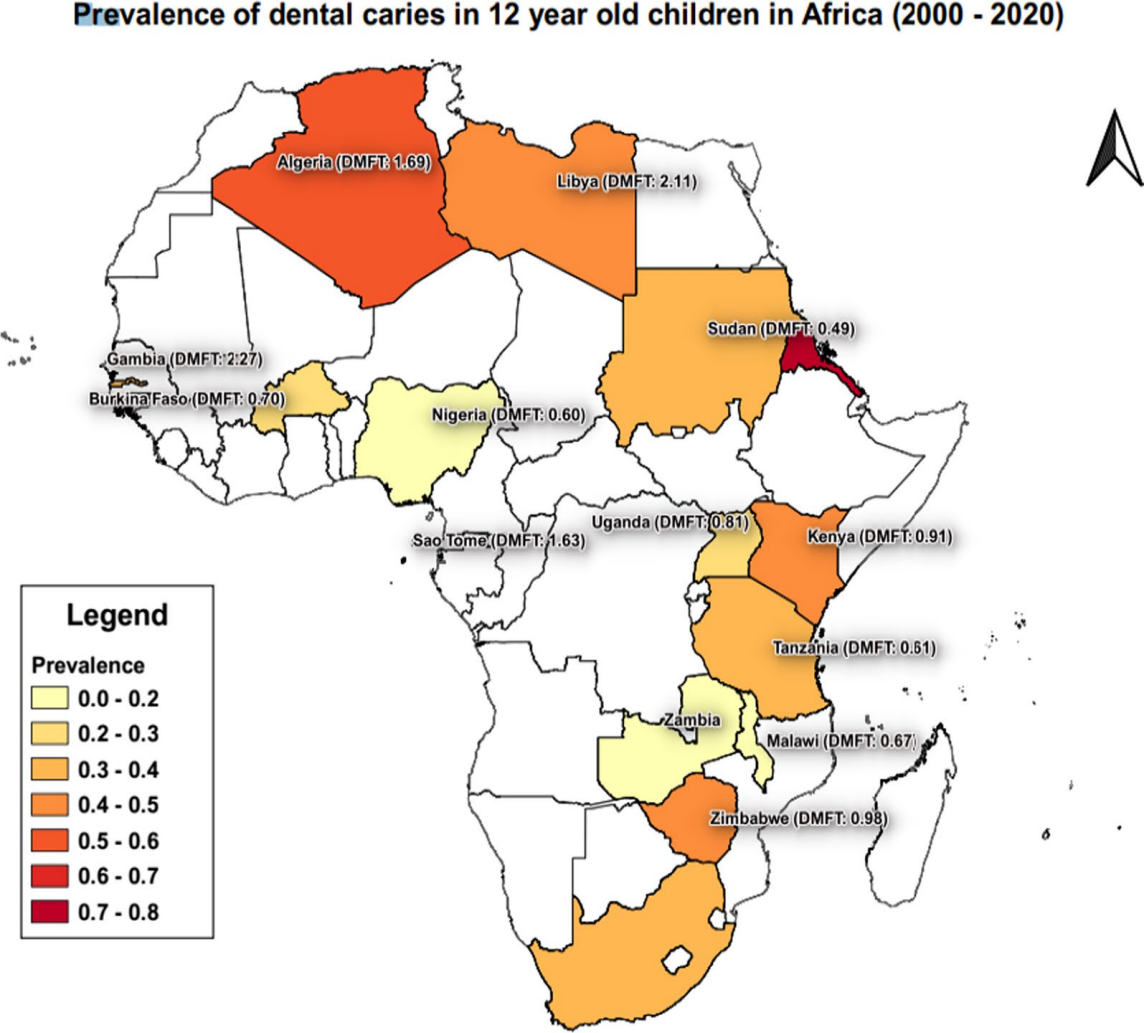
Distribution of mean prevalence across the continent

**Fig. 3:**
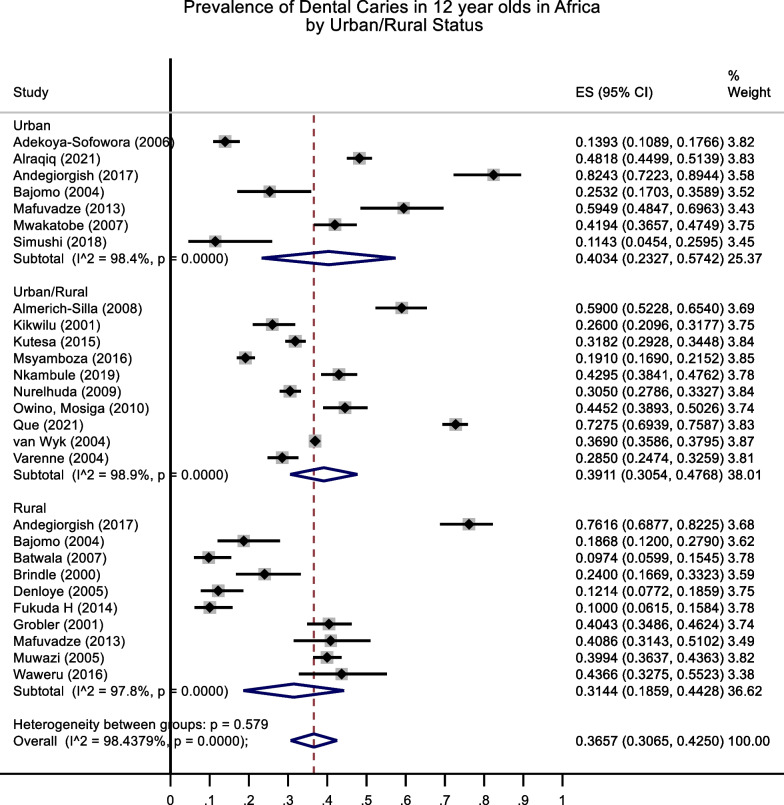
Meta-Analysis of prevalence of dental caries for urban and rural settings

A total sample size of 18,080 participants were included in this review. Table 3 summarizes the findings from the included studies. There were 22 articles that determined prevalence and 21 articles which could be included for the DMFT meta-analysis. The overall prevalence was 36% (29.4–41.7) (Fig. 2). The overall mean DMFT was 1.09 (0.91–1.3) [4]. All the studies, except two, utilized the World Health Organization (WHO) criteria (III, IV or V) for the dental examination. The rural prevalence (31%) was lower than the urban prevalence (40%) Fig. 3.

**Table 3:** Study characteristics

Author	Age	Country	DMFT mean (SD)	DT mean (SD)	MT mean (SD)	FT mean(SD)	n (caries)	N (Total))	Setting	Sex	SES	Data Collection Period	Diagnostic criteria	Sampling	Prevalence (5)
Adekoya-Sofowora,	12	Nigeria	0.14				56	402	School	349 ♂ 153 ♀	NA	2003	WHO 4th Ed	Random selection	13.93
2006 [77]															
Almerich-Silla,	11–13	Algeria	1.69 [2]	1.07 (1.78)			125	212	Refugee Camps	No detail	NA	2007	WHO 4th Ed	Convenience sampling technique	58.96
2008 [78]															
Alraqiq [12]	12	Libya	1.7 (1.6)				450	934	school based	419 ♂ 514♀	NA	2019	Association of State and Territorial Dental Directors	Not stated	48.18
Andegiorgish [10]	12	Eritrea	2.5 (2.21)	2.44 (2.13)	0.05 (0.27)	0.01 (0.2)	176	225	school	81 ♂ 95 ♀	NA	2017	WHO 5th ed	Random selection	78.22
Bajomo [79]	12	South Africa	0.61(1.5)	0.55(1.33)	0.02(0.19)	0.04(0.07)	37	170	school	18 ♂ 19 ♀	NA	2004	WHO 4th Ed	Stratified Random Sampling	21.76
Batwala [80]	11–12	Uganda					15	154	school-based survey	♂DMFT: 1.3 ± 0.7	NA	2006	WHO 3rd	Stratified Two-stage cluster sampling	9.74
										♀DMFT =					
										1.6 ± 0.8					
Braimoh [81]	12–15	Nigeria	0.21 (0.6)					66				2013	WHO 5th ed	Not stated	
Brindle [82]	12	South Africa	0.4 (0.4)				24	100	Dental Practices(private and public)/ Schools/households		NA	1999	WHO 3rd	Not stated	24.0
Chukwumah [83]	12	Nigeria	1.76 (1.72)	2.07(1.46)	0.00(0.0)	0.00(0.00)	0	0	School-based study	No detail	NA	2015		Random selection	
Denloye [36, 84]	12	Nigeria	1.94 (1.0)				17	140	School-based study	7 ♂	NA	2003	WHO 4th Ed	Not stated	21.76
										10 ♀					
Elfseyie [85]	12	Libya	2.66 (0.21)					50	Dental Faculty	No detail	NA	2019	WHO 3rd	Not stated	
Fukuda [86]	12	Kenya	0.24				15	150	School-based study	58 ♂ 92 ♀	NA	2011	WHO 4^th^ Ed	Not stated	10.0
Grobler [50]	12	South Africa	1.74 (0.3)				114	282	School-based study	No detail	NA	2000	WHO 3rd	Not stated	40.43
Kikwilu [87]	12	Tanzania	0.46 (0.96)				65	250	School-based study	No detail	NA	2000	WHO 3rd	Not stated	26.0
Kosovic [88]	12	The Gambia	2.27(2.31)				52	172	School-based study	87♂	NA	2000	WHO 4th Ed	Random selection	30.23
										85 ♀					
Kutesa [89]	11–13	Uganda	0.73				391	1230	School-based survey	No detail	Low SES	2014	WHO 5th ed	Multi-stage sampling technique	31.79
Mafuvadze [90]	12	Zimbabwe	0.975	1.2 urban; 0.65 rural	0.05 urban; 0.01 rural	0.01urban; 0.00 rural	85	172	School-based study	74 ♂	low SES	2012	WHO 4th Ed	Not stated	49.42
										98 ♀					
Molete [91]	11–12	South Africa	1.8 (2.3)					181	School-based survey	No detail	unemployment rate of 24.2	2013	WHO 5th ed	Multi-stage sampling technique	
Msyamboza [92]	12	Malawi	0.67	0.41	0.26	0	211	1115	home based	541 ♂	NA	2013	WHO 5th ed	Not stated	18.92
										574 ♀					
Muwazi [93]	11.5–12.5	Uganda	0.9	87.80%	11.30%	0.90%	277	696	School-based study	328 ♂ 368 ♀	NA	2002	WHO 3rd	Not stated	39.8
Mwakatobe [94]	12	Tanzania	0.76 (1.17)				129	310	School-based survey	175 ♀ (56.5%) and 135 ♂ (43.5%); (DT)		2003	WHO 4th Ed	Not stated	41.61
										Component was higher in girls (48%) than in boys					
										(33.3%)					
Nkambule [95]	12	South Africa	1.19 (1.79)	1.1 (1.59)	0.0 (± 0.25)	0.1 (± 0.45)	189	440	School-based study	207 ♀ 233 ♂	All	2017	WHO.Oral health surveys: basic method	Not stated	42.95
Nurelhuda [96]	12	Sudan	0.49 (1.06)	0.43 (0.97)	0.03 (0.23)	0.03 (0.25)	338	1109	School-based survey	No detail	35.4% middle income	2007	WHO 3rd	Not stated	30.48
Okoye [97]	12	Nigeria	0.59 (1.11)					51	School-based survey	20 ♂ (dmft = 0.4(0.88);	NA	2010	WHO 4th Ed	Stratified Random Sampling	
										31 ♀ (0.71(1.24)					
Owino [98]	12	Kenya	0.92 (1.36)				130	292	School-based study	140 ♂ 152 ♀	NA	2010	WHO 4th Ed	Not stated	44.52
Que [16]	9–11	Sao Tome	1.63 (1.51)				526	723	School-based study	No detail	NA	2020	WHO 5th ed	Not stated	72.75
Smit [99]	12	South Africa	2.4							No detail	NA	2015	WHO 4th Ed	Not stated	
van Wyk [100]	12	South Africa	1.1	0.8	0.2	0.1	8226	303,594	National survey	No detail	NA	1999	WHO 3rd	Not stated	2.71
Varenne [101]	12	Burkino Faso	0.7 (1.35)	0.7	0.01	0	143	505	Household survey		NA	2003	WHO 3rd	Multi-stage sampling technique	44.52
										267 ♂ 26.6%; DMFT = 0.6 238 ♀ 30.7%; DMFT = 0.8					
Waweru (102)	12–15	Kenya	1.66 (2.66)	1.62	0.04	0	31	71	School-based study	25 ♂ 46 ♀	NA	2015	WHO 5th ed	Random selection	43.66

*NA* Not available; *SES* Socio-economic status; *DMFT* Decayed, Missing, Filled teeth; *DT* Decayed teeth; *MT* Missing teeth; *FT* Filled teeth; *WHO* World health Organization

♂-Males

♀-Females

The overall prevalence of dental caries in 12-year -olds in Africa was 36% (29.4–41.7%) (Fig. 2). The highest prevalence was recorded in Eritrea in 2017 (78%; 95% CI 72.4–83.1%), followed by Sao Tome (73%, 95% CI 69.4–75.9%) with the lowest prevalence scores in Zambia (11%) and Nigeria (13%) (Table 4). Overall, the confidence intervals were relatively large indicating small sample sizes and a wide range within the results.

**Table 4:** Prevalence of caries and DMFT per Country

Country	Prevalence (95% CI)	No of articles	DMFT (95% CI)	No of articles
Algeria	59 (0.523–0.654)	1	1.69 (1.463–1.917)	1
Burkino Faso	29 (0.247–0.326)	1	0.70 (0.639–0.761)	1
Eritrea	78 (0.724–0.831)	1	2.50 (2.173–2.827)	1
Kenya	33(0.066–0.584)	3	0.91 (0.295–1.525)	3
Libya	48 (0.45–0.514)	1	2.11 (1.178–3.038)	2
Malawi	19 (0.168–0.214)	1	0.67 (0.631–0.709)	1
Nigeria	13 (0.106–0.163)	2	0.60 (0.372–0.833)	4
Sao Tome	73 (0.694–0.759)	1	1.63 (1.509–1.747)	1
South Africa	34 (0.276–0.401)	5	1.13 (0.795–1.454)	6
Sudan	31 (0.279–0.333)	1	0.49 (0.461–0.519)	1
Tanzania	34 (0.299–0.376)	2	0.61 (0.314–0.902)	2
The Gambia	30 (0.239–0.375)	1	2.27 (1.931–2.609)	1
Uganda	27 (0.126–0.418)	3	0.81 (0.646–0.979)	3
Zambia	11 (0.045–0.26)	1		1
Zimbabwe	49 (0.42–0.568)	1	0.98 (0.829–1.121)	1
Overall	36 (0.294–0.417)	25	1.09 (0.914–1.266)	28

The mean DMFT score was the highest for Eritrea (2.5 (95% C.I.: 2.17–2.88)) followed by The Gambia (2.27 (95% C.I.: 1.93–2.61)) and Libya (2.11 (95% C.I.: 1.18–3.04)).

All of the studies were cross sectional in design and carried out by oral health personnel including dentists and dental therapists.

### Diagnostic criteria is the method used to evaluate dental caries

The overall DMFT was 1.09 (95% C.I.: 0.91–1.27) (Table 3). The lowest DMFT scores were recorded in Sudan (0.49 (95% C.I.: 0.46–0.52)), Nigeria (0.60) and Burkina Faso (0.70 (95% C.I.: 0.37–0.83)) while the highest scores were obtained in Eritrea (2.50 (95% C.I.: 2.17–2.83)), the Gambia (2.27 (95% C.I.:1.93–2.61)) and Libya (2.11 (95% C.I.: 1.18–3.04)). In general, the confidence intervals were relatively large indicating small samples and a wide range within the results.

Urban cities had the highest pooled prevalence (40%, 95% C.I.: 23.3–57.4%) compared to rural cities (31%, 95% C.I.: 18.6–44.3%) (Fig. 3). Some studies did not indicate the setting and were pooled into a single group called urban/rural.

**Fig. 4:**
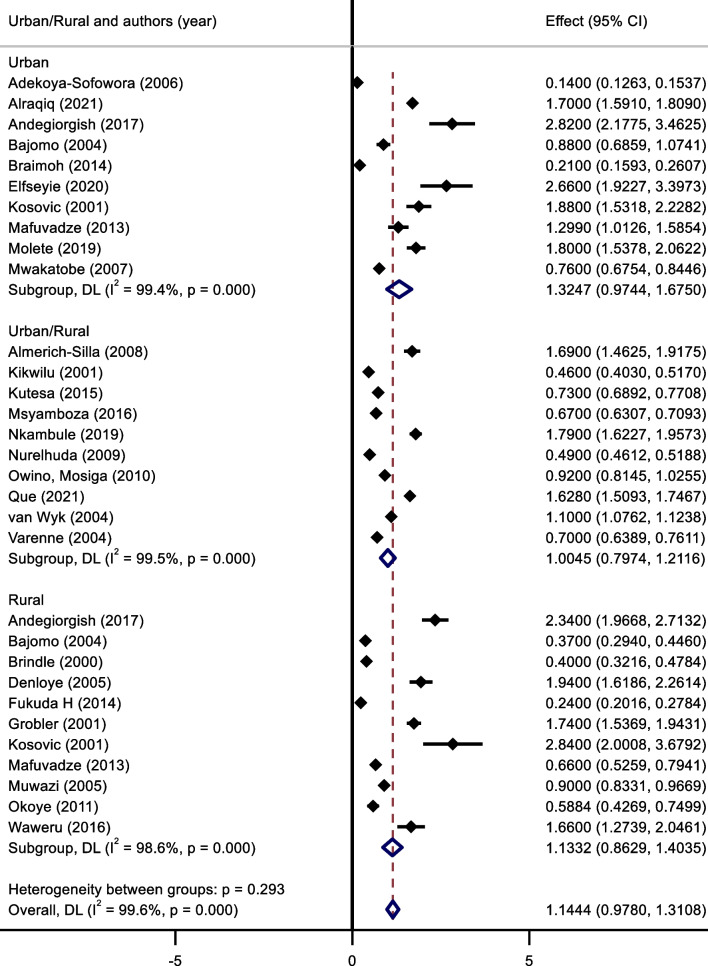
Meta-Analysis of mean DMFT scores for urban/rural settings.* Note* Weights and between-subgroup heterogeneity test are from random-effects model

The mean DMFT in rural and urban settings were similar to the caries prevalence; urban settings recording a mean DMFT score of 1.32 (95% C.I.: 0.97–1.68) and rural settings recording a lower 1.13 (95% C.I.: 0.86–1.4) score (Fig. 4). Those studies which did not indicate the type of setting, reported a mean score of 1.00 (95% C.I.: 0.80–1.21. Again, similar to the other results, the confidence intervals were relatively large.

**Fig. 5:**
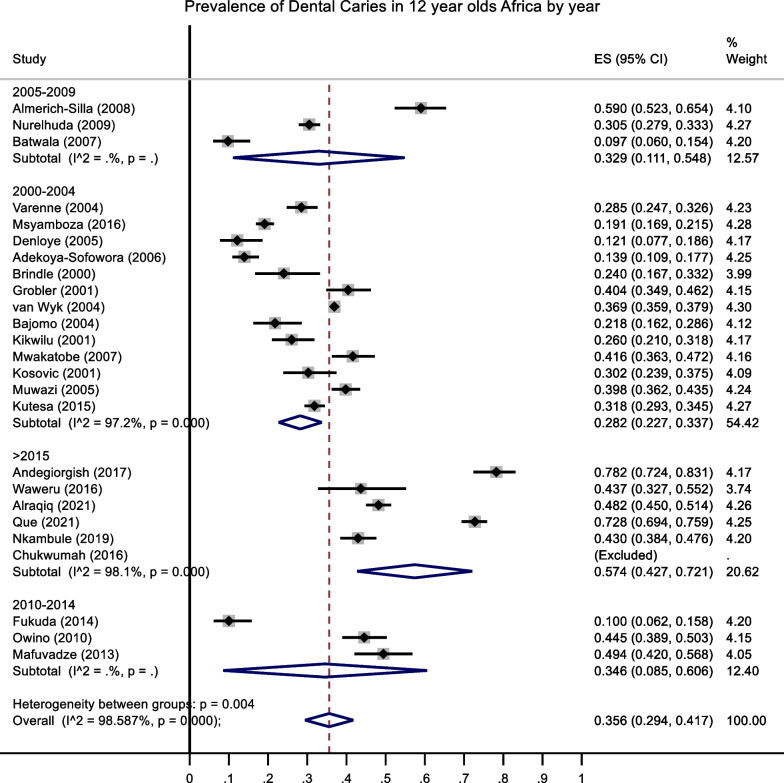
Meta-Analysis of prevalence of dental caries for different time periods

**Fig. 6:**
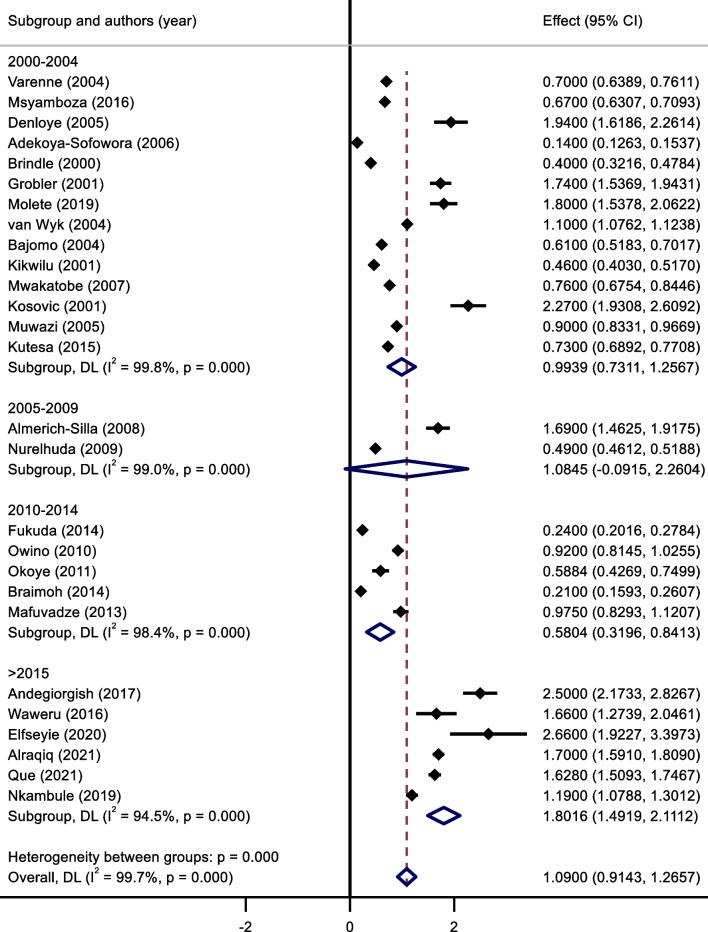
Meta-Analysis of DMFT scores during different time periods. NOTE: Weights and between-subgroup heterogeneity test are from random-effects mod

The prevalence of dental caries in 12-year-old children in 2000–2004 was 28.2% (95% C.I.: 22.7–33.7), 2005–2009, 32.9% (95% C.I.: 11.1–54.8), 2010–2014 was 34.6% (95% C.I.:8.5–60.6) and in 2015 and above, it was 57.4% (95% C.I.:42.7–72.1) (Figs. 5 and 6). Although the confidence intervals were quite large, nevertheless the DC increased over time.

The mean DMFT scores was 0.99 (95% C.I.:0.73–1.26) in 2000–2004, 1.08 (95% C.I.:0.0–2.26) in 2005–2009, 0.58 (95% C.I.:0.32–0.84) in 2010–2014, and increased to 1.80 (95% C.I.:1.49–2.11) from 2015 to present (Fig. 6).

### Pooled effect size of dental caries severity in Africa

The pooled effect size of dental caries severity was 1.09 (95% C.I .:0.914–1.27) (Fig. 3). The highest mean DMFT score was seen in Eritrea (2.5, 95% C.I.: 2.17–2.83), and the lowest was seen in Tanzania (0.61, 95% C.I.: 0.31–0.91). Urban cities had the highest mean DMFT score (1.32, 95% C.I.: 0.97–1.68), compared to rural cities (1.13, 95% C.I.: 0.86–1.4) (Fig. 5).

### Critical appraisal

Twenty-eight studies found that the sampling frame was appropriately addressed to the target population and that the study participants were sampled in an appropriate way. In addition, 28 articles found valid methods to have been employed to identify dental caries. However, the study subjects were not described in detail in 22 studies, there was not a sufficient sample size for 23 of the studies and 18 studies found that appropriate statistical analysis was not used (Table 5).

**Table 5:** Critical appraisal according to the Joanna Briggs Institute (JBI) criteria

Author	Was the sample frame appropriate to address the target population?	Were study participants sampled in an appropriate way?	Was the sample size adequate?	Were the study subjects and the setting described in detail?	Was the data analysis conducted with sufficient coverage of the identified sample?	Were valid methods used for the identification of the condition?	Was the condition measured in a standard, reliable way for all participants?	Was appropriate statistical analysis used?	Was the response rate adequate, and if not, was the low response rate managed appropriately?	Total
Adekoya-Sofowora [77]	1	1	1	0	0	1	0	0	0	4
Almerich silla [78]	1	1	1	1	1	1	1	1	1	9
Alraqiq [12]	1	1	1	1	1	1	1	1	1	9
Andegiorgish [10]	1	1	1	1	1	1	1	1	1	9
Bajomo [79]	1	1	1	1	1	1	1	1	1	9
Batwala [80]	0	1	1	0	0	1	0	1	1	5
Braimoh [81]	1	1	0	1	1	1	1	0	0	6
Brindle [82]	1	1	1	1	1	1	1	1	1	9
Chukwumah [83]	1	1	1	1	0	1	1	1	0	7
Denloye [84]	1	1	1	1	1	1	0	0	1	7
Elfseyie [85]	1	1	1	0	1	1	1	1	1	8
Fukuda [86]	1	1	0	0	0	0	1	1	1	5
Grobler [50]	1	0	0	1	1	1	1	0	0	5
Kikwilu [87]	1	1	1	0	0	0	1	0	1	5
Kosovic [88]	1	0	0	0	1	1	1	0	0	4
Kutesa [89]	1	1	1	1	1	1	1	0	1	8
Mafuvadze [90]	1	1	0	1	1	1	0	0	0	5
Molete [91]	1	1	1	1	1	1	1	1	1	9
Msyamboza [92]	1	1	1	1	1	1	0	0	0	6
Muwazi [93]	1	1	1	1	1	1	1	0	1	8
Mwakatobe [94]	1	1	1	1	1	1	1	1	1	9
Nkambule [95]	1	1	1	1	1	1	1	0	1	8
Nurelhuda [96]	1	1	1	1	1	1	1	1	1	9
Okoye [97]	0	1	0	0	0	1	0	1	1	4
Owino [98]	1	1	1	1	1	1	1	1	1	9
Que [16]	1	1	1	1	0	1	1	1	0	7
Smit [99]	1	1	1	1	0	1	0	0	1	6
van Wyk PJ [100]	1	1	1	1	1	1	1	1	1	9
Varenne [101]	1	1	1	1	1	1	1	1	1	9
Waweru [102]	1	1	0	0	1	1	0	1	0	5

### Publication bias

**Fig. 7:**
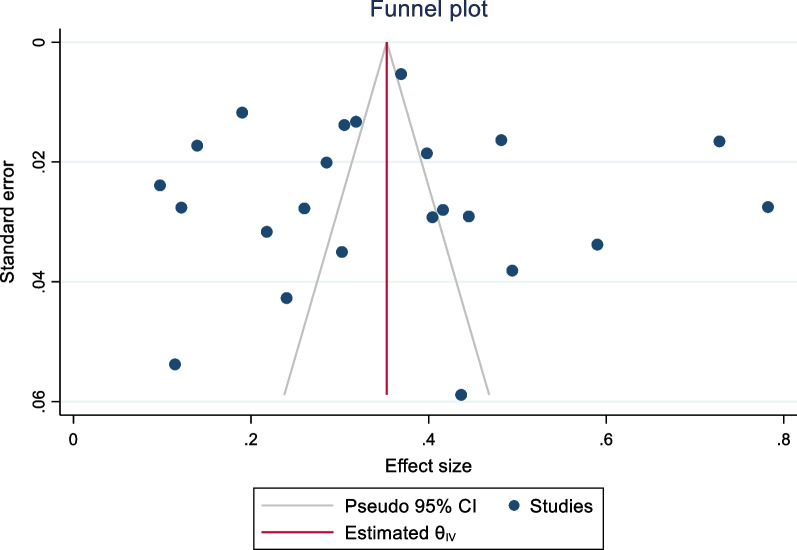
Publication bias for prevalence

**Fig. 8:**
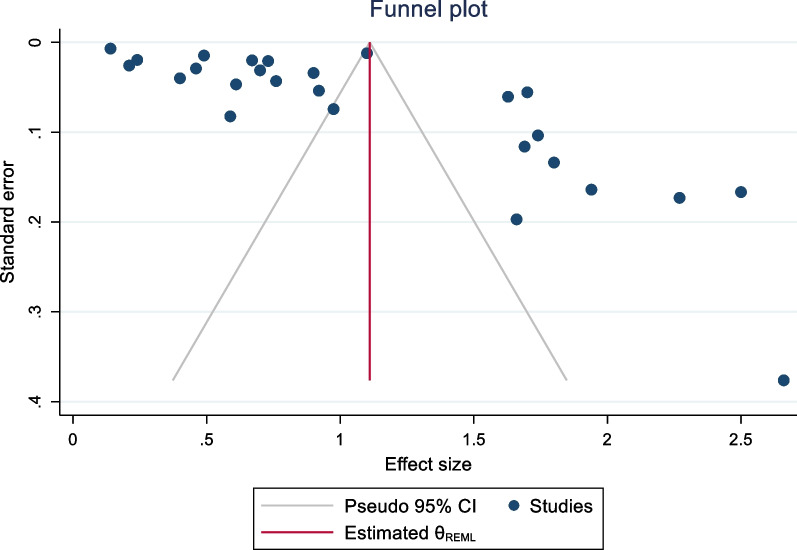
Publication bias for dmft

Begg’s test and funnel plots were both significant which indicates that there was publication bias (*p* < 0.001). Publication bias was also assessed using Duval and Tweedies “Trim and Fill” method for prevalence (Fig. 7) and DMFT scores (Fig. 8). The prevalence was 35.4 (34.8–36.1), which indicated that zero studies were missing using a random effects model. Furthermore, the DMFT score was 0.47 (0.46–0.48), and indicated that 6 studies were missing. These results indicated that there was publication bias for the DMFT outcome but not for prevalence.

The critical appraisal was done using the Joanna Briggs Institute (JBI) criteria and a maximum score of 9 indicated that there was minimal bias.

## Discussion

This current study is the first of its kind evaluating the prevalence of DC in Africa for children of 12 years of age. The 12-year old age group is often neglected with many studies focussing on either the under 6-year olds and/or the 15-year olds. The 12-year-olds provide an ideal window to measure the impact of school based and fissure sealant programmes which are often undertaken when children are between the ages of 6 and 8 years old. In addition, the 12-year- olds also provide an opportunity to assess the status of the permanent teeth and the exfoliation of the primary dentition.

The current prevalence of DC was 36% (95% C.I.: 29.4–41.7%) which was lower than a study on 12-year -olds in Puerto Rico (39.3%) [103]. However, the caries prevalence was higher than that recorded on 12-year-old children in Haiti (31%) [104]. This discrepancy might be due to the differences in socio-economic status, the data collection indices, the educational levels and fluoride levels. These studies were individual studies while the current study is a systematic review of all African countries which could explain the difference in the results.

The mean DMFT was 1.09 which was much lower than the WHO goal of 12-year-old’s which estimates that the DMFT score be less than 3 [105]. Interestingly, the DMFT and caries prevalence was the highest in Eritrea (2.50 and 78% respectively). Eritrea has had many challenges including famines, recurrent wars and drought which has resulted in over 66% of the population living below the poverty line [106]. These factors could have impacted on the provision of dental services and education in the early years of these children which may have contributed to the high caries prevalence. The impact of war could have also decreased access to essentials such as toothbrushes and toothpaste which could have been partly responsible for the caries scores. Although Vasireddy [18]. reported that poverty could have a negative impact on dental caries prevalence, the impact of famine and war could have easily been responsible for the high caries scores.

The other country with a relatively high mean DMFT score was Libya (2.11). The study in Libya with the highest mean DMFT score was 2.66 and a possible reason for this was the setting. This study was carried put at a paediatric dental hospital and not a school setting. As a result, all those who attended required some sort of dental treatment and this could be the reason for the high score. This was confirmed by the second Libyan study which reported a mean DMFT score of 1.70 which could indicate the actual prevalence to be slightly lower.

The Gambia also recorded a high mean DMFT score (2.27). These results showed that rural children had a significantly higher mean DMFT score (2.84) compared to high socio-economic status urban children (1.69). The possible reasons for this high score was cited as a lack of access to services, poor diet and poor dental knowledge among young children. Preventive programs and educational programmes need to be implemented in general in African countries, especially in rural areas.

Countries with the lowest mean DMFT scores were in Sudan (0.49), Nigeria (0.60) and Burkina Faso (0.70). The Nigerian studies all cited the lower DMFT scores to the school programs, frequency of dental visits and brushing frequencies. This showed that a good environment can assist in the reduction of dental caries. The Sudanese study, although having a low mean DMFT score, reported that urban children and children of a high socioeconomic status had higher mean DMFT scores compared to rural children. The low mean DMFT score amongst the rural cohort was attributed to the diet and high level of oral hygiene.

A subgroup analysis showed that urban status resulted in a higher caries prevalence compared to rural status. This finding was corroborated by Al-Akwa [107] who also found that caries prevalence was higher in urban areas compared to rural areas. Urban poverty has also been linked to poor access to healthy eating and food insecurity [108]. The mean DMFT scores were also higher in urban cities compared to rural cities. This could be due to the diet in urban areas which usually comprise of refined carbohydrates rather than the rural diets which usually contain less sugars. It’s also possible that communities in rural areas utilise borehole water which tends to contain more fluoride compared to the urban areas which have a central water source that usually is derived from dams. The water derived from dams often has a much lower fluoride concentration.

Although the caries prevalence changed over time, the changes were not significant. It was expected that the caries prevalence would have decreased over time, but the results showed that the prevalence steadily increased. This could have been due to urbanization, access to refined carbohydrates, increase sugar intake and possibly a more urbanised diet compared to a more rural diet.

Twenty seven of the thirty articles presented with a critical appraisal of more than 50%. Eleven articles presented with a score of nine (Table 5).

## Conclusions and recommendations

The mean DMFT and caries prevalence reported in this systematic review demonstrates that the WHO goal for 12-year-olds has been reached in Africa. We should however view these findings with great care because of the high heterogeneity between the studies and the high risk of bias. We would recommend that studies of high quality be conducted in Africa so that we can determine the mean DMFT scores and caries prevalence. Studies should include the setting of the sample (rural or urban) the socio-economic status, the indices used, the calibration of examiners, the appropriate statistical test and sample size and oral hygiene practices. This will allow studies to be pooled and compared to identify possible programmes that could impact on the dental disease burden.

### Limitations

Many studies did not include all the data and in some of the studies, the data was collapsed and difficult to identify. This made the analysis more difficult. Studies that were not published in English were excluded as there is a great diversity in languages in Africa.

Given the different countries that were included, the times at which the data was collected and the settings, there was no inter-country calibration and as such the results need to be interpreted with caution.

## Data Availability

The datasets for this study can be made available on reasonable request to fkimmie@uwc.ac.za.
